# End-to-side neurorraphy with and without perineurium

**DOI:** 10.1590/S1516-31802002000300009

**Published:** 2002-05-02

**Authors:** Orestes Barini

Interest in the technique of "endto-side neurorrhaphy and side-to-side neurorrhaphy" has been reactivated consequent to the presentation of several papers on this topic at a Congress that took place recently in Botucatu, São Paulo. Motivated by this, I would like to present a critical analysis of the article published in the "São Paulo Medical Journal", Vol. 116, Sep/Oct 1998, from Fausto Viterbo et al., who are great defenders of this concept.

## Discussion

The exposition provided in their work presents several errors, which I have addressed in the following series of points:

1. The title uses the word "perineurium" wrongly, referring to the peripheral layer of the nerves, whose correct name is "epineurium": perineurium is the name of the sheath that involves a group of axons. In the abstract of this same work, the correct name, epineurium, is used.
2. Because of the wrong denomination used for the nerve sheath, which is presented as "perineurium", the pictures nos. 4, 5 and 6 document an impossible surgical intervention: the extirpation of the perineurium, the sheath involving a group of axons in the nerve fibers.
3. Figures 4, 5 and 6 have legends indicating the comparison between two surgical groups "without perineurium" wrongly, since one of them, shown on the left, corresponds to surgeries conducted on the right side, according to the text, with the epineurium remaining untouched.
4. Some of the details in the exposition are incomplete. Thus, what is the purpose of inserting the proximal stump of the peroneal nerve as an implantation in the abductor muscle? What is the purpose of sectioning the cutaneous caudal nerve of the calf, which is done concomitantly to the sectioning of the tibial nerve, located distally to the position of the neurorrhaphy? There is no representation in Figure 2 of the sectioning of the peroneal nerve, located distally to the position of the neurorrhaphy that is indicated in the text.
5. No presentation was made of the transverse section of the peroneal nerve, located distally to the neurorrhaphy, which was said to have shown "many regenerated fibers". Such a presentation would have great documentary importance.
6. More importantly, there is a lack of information about the functional ability of the tibial-cranial muscle ("TCM") that is enervated by the peroneal nerve, six months after the time of the neurorrhaphy. Such information is of capital importance for evaluating the efficiency of the neurorrhaphy that was done.
7. Figure 3, the only documentation presented in relation to this matter of controversy, does not show an end-to-side neurorrhaphy that plainly corresponds to the respective title of the article (although the respective legend refers to an endto-side neurorrhaphy), but rather a side-to-side one. The figure shows nothing more than the remains of nerve fibers in one of the two coupled nerves, in each half of the figure. These ought to be the receptor nerves, especially in the left half of the figure. In this way, the legend would correspond to the information that the right half would be the documentation of a side-to-side neurorrhaphy of the receptor nerve, without epineurium.
8. With regard to the "Results" section: the statement about supposed histological changes to the nerves, six months after neurorrhaphy, according to which longitudinal cuts taken from the area of the neurorrhaphy would *"suggest"* that the epineurium and perineurium had disappeared and lateral sprouting had occurred, and which Figure 3 documents, *is the absolute opposite of the reality*. Figure 3 corresponds to a side-to-side neurorrhaphy: the borders between the two nerves in each half of the figure are quite clear, and there is a tenuous space between the majority of the neurorrhaphy components. One further detail: in the right half of the photograph, there are more distinctive tortuous traces, coming from the connective tissue. It is particularly important that no lateral sprouting takes place over there.
9. In the account regarding nerve fibers in the specimens taken six months after the neurorrhaphy, from the distal portions of the peroneal nerve and the proximal and distal portions of the tibial nerve, in relation to the position of the neurorrhaphy, the information given is that a number of fibers are shown to be conserved in the nerves pre and post- neurorrhaphy. An explanation is nonetheless merited for the noticeable loss of fibers from the tibial nerve portion that is the donor nerve, from 939 to 755 on the left side of the post-neurorrhaphy figure. See also my criticism of the nerve fiber concept, in the Conclusion section, below.
10. The information regarding weight, cytometry and electrophysiology of the tibial-cranial muscle ("TCM muscles") shows the conservation of these characteristics, which therefore do not depend on the intervention.
11. The information commented on in items 7, 8, 9 and 10 of this letter does not make a comparison between the situation before and after the neurorrhaphy, but only between surgery done with and without opening up the epineurium of the receptor nerve. Thus, such information does not prove the hypothesis of the "energization" of nerves.

## CONCLUSION

The corroboration of the author's "discovery" should be based on some epilog: on documentation that gives evidence that the denervated muscle, "energized" by the neurorrhaphy, was fully functional. And this was not done.

Following this, the histological conservation of the "energized" nerve should be corroborated.

It is not enough just to provide an image obtained with a 90 times magnification showing the nerve fibers. With such a magnification, it is not possible to document the existence of the axon. The component accepted by the author of the article as a "nerve fiber" does not have a visible axon, and without an axon, no functional nerve conduction can be presumed.

The presentation of images with greater magnification, showing the presence of axons in the "energized" nerve fibers, would unquestionably and undeniably be effective (rather than the image with 90 times magnification, used for obtaining Figure 3). And this could be obtained via the same staining process, the Regand hematoxylin.

With regard to the results from "energizing" a sectioned nerve, using the neurorrhaphy that the author proposed, the consequences would not be as the author expected and indicated.

The motor action of the "energized" nerve would take place *concomitantly* with the motor action of the donator nerve, triggered by action from the motor area of the cerebral cortex in the donor nerve. The motor action of the motor cerebral area in the receptor nerve would be blocked, because of the sectioning of this nerve, associated with secondary degeneration of its axons. Neurorrhaphy of the facial nerve sectioned with the hypoglossal nerve, for instance, would result in the contraction of the facial musculature, unleashed by the movement of the respective hemilingual structure. There would be tonus in the facial musculature, but not mimic movement.

Concomitantly, sensitivity in a skin area corresponding to an "energized" sensitive nerve would never reach the corresponding cerebral sensitive area, because its axons, albeit "energized", would be interrupted in the area of the neurorrhaphy. That is, unless the "energized" axons in the receptor nerve could also "energize" the sensitive axons in the donor nerve, in which case the sensitive stimuli would reach the cerebral sensitive area of the donor nerve. Otherwise, the patient would continue with anesthesia of the skin area corresponding to the receptor nerve, and would feel sensitive stimuli in the skin area corresponding to the donor nerve.

