# Supplementary material for: End-to-side neurorraphy with and without perineurium
Source: Sao Paulo Med J. 2002 May 2;120(3):93–5. doi: 10.1590/S1516-31802002000300009 (PMC11151437; doi:10.1590/S1516-31802002000300009)
Supplement: Related texts

# REPLY-LETTER TO EDITOR

**Authors:** Fausto Viterbo

**Answer to Dr Orestes Barini**

First of all, I would like to thank Dr Orestes Barini for his detailed comments on our article.

This new way of connecting peripheral nerves has permitted various experimental studies and opened up countless therapeutic possibilities, many of them already in clinical use.

As with all recent events in medicine, some arouse admiration, others skepticism. Several of my colleagne's observations are well reasoned and we respond thus:

1. In reality, we did remove both the epineurium and perineurium. As it is obvious that we needed to remove the epineurium in order to remove the perineurium, and to keep the title to a reasonable length, we preferred to use just perineurium in the title. A more complete Abstract text would have been "Epineurium and Perineurium Removal". Other authors have also used this shorter form.
2. Perineurium removal is not only perfectly possible, but we have performed it, and so have other authors.
3. The word "without" on the left side of the graphs was copied incorrectly. This has been corrected to "with". Thank you.
4. The objective of implanting the proximal end of the peroneal nerve into the abductor muscle was to avoid the possibility of motor contamination of this segment in the distal ending of the same nerve, which would have made the work impracticable. This detail has obviously been highlighted since our early works and in other publications. The second occurrence of the legend for Figure 2 is incorrect. We agree that we should have had another drawing of the peroneal nerve section. What is clear and obvious to the author is not necessarily clear to those not accustomed to these studies.
5. We agree that a photograph of a cross-section of the nerve fibers would have been better.
6. Functional evaluation was not the objective of this study.
7. End-to-side was performed, not side-to-side. As we could not have tension in the donor receptor nerve, we always used end-to-side with the receptor nerve for much longer than necessary. Thus, it was common for us to find the receptor nerve laterally attached to the donor nerve for a short length.
8. Even though the photographic documentation was inadequate for showing the lateral sprouting, we had no doubt as to its occurrence. This had been extensively proven in our earlier works and in those of other colleagues.
9. It was not the objective of this work to analyze the number of donor nerve fibers pre and post-neurorrhaphy. Other works have analyzed this.
10. We do not agree. The effect of reinnervation by end-to-side had been well substantiated in our earlier works and in those of other colleagues.
11. The single objective of this work was to verify the effect on the epineurium-perineurium from end-to-side. Our earlier work, and other authors’ work, had analyzed and verified the efficiency of endto-side in muscular reinnervation and the restoration of sensitivity.

As for the brain's incapacity to identify end-to-side reinnervated areas in different nerves and antagonistic muscles, I believe that my colleague has underestimated this organ. Only time will tell.

I take this opportunity to mention that I have just returned from participating in the "50 Years of Peripheral Nerve Surgery" Congress in Vienna on March 14–16, organized by the World Federation of Neurosurgical Societies, at which I was presented with The Hanno Millesi Award for major contributions in the area of peripheral nerve microsurgery over the last five years. Our work on endto-side made us worthy of this honor.

In closing, I would like to make it clear that our first publication in this area was in the "São Paulo Medical Journal", and this article has been referred to by many authors from different countries. This reinforces my opinion that this important medical journal must keep its doors open to experimental works, as has been its editorial policy.

Finally, I would once more like to express my thanks for my colleague's interest in our work.

Best regards

## ERRATUM

In the article "**Acute abdomen due to late retroperineal extravasation from a femoral venous catheter in a newborn**", published in the edition dated 7 March 2002, volume 120, number 2, page 59, authored by Prof. Jaques Sztajnbok and Eduardo Juan Troster, the correct name of the Institution is Hospital Nossa Senhora da Penha and not Our Lady of Penha Hospital.
